# Development and evaluation of mPEG-PLLA polymeric micelles encapsulating enrofloxacin for enhanced solubility, bioavailability, and antibacterial performance

**DOI:** 10.3389/fvets.2025.1595137

**Published:** 2025-07-16

**Authors:** Yanling Sun, Yanan Mao, Xin He, Xinghua Zhao

**Affiliations:** College of Veterinary Medicine, Hebei Agricultural University, Baoding, China

**Keywords:** enrofloxacin, MPEG-PLLA, micelles, oral bioavailability, antibacterial activity

## Abstract

The aim of this study was to prepare polymeric micelles composed of enrofloxacin (ENR) and methoxy poly (ethylene glycol)-poly(lactide) (mPEG-PLLA) using a solvent evaporation method to overcome the solubility-limited oral bioavailability of ENR. The formulation was optimized using a Box–Behnken design (BBD) to obtain ENR polymeric micelles (ENR-m) with high drug loading (DL, %) and entrapment efficiency (EE, %). The physicochemical properties, *in vitro* drug release, pharmacokinetics, and antibacterial efficacy were evaluated in comparison to pure ENR. ENR-m was successfully prepared and demonstrated satisfactory drug loading (68.38 ± 0.22%), entrapment efficiency (88.40 ± 0.91%), particle size (PS) (133.67 ± 3.10 nm), and polydispersity index (PDI) (0.13 ± 0.03). The ENR-m also exhibited excellent stability under environmental conditions (40°C and 75% relative humidity (RH)). *In vitro* release of ENR from micelles was accelerated in a PBS solution. A pharmacokinetic study on beagles revealed that the oral bioavailability of ENR-m was enhanced by approximately 1.60-fold compared to pure ENR (*p* < 0.01) and by 1.66-fold compared to commercially available tablets of ENR (*p* < 0.01). The antibacterial activity of ENR-m against *Escherichia coli* (*E. coli*) and *Salmonella typhi* (*S. typhi*) was stronger than that of pure ENR.

## 1 Introduction

**SCHEME 1:**
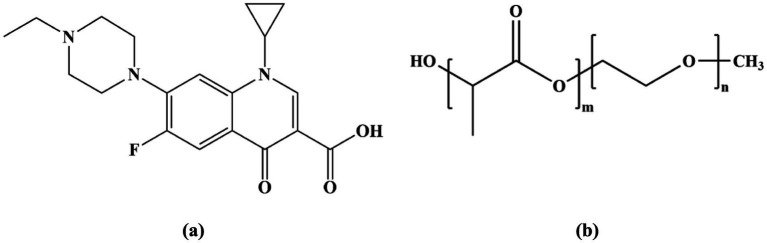
Structures of **(A)** ENR and **(B)** mPEG-PLLA.

Enrofloxacin (ENR), chemically known as 1-cyclopropyl-7-(4-ethyl-1-piperazinyl)-6-fluoro-1,4-dihidro-4-oxo-3-quinoline carboxylic acid (Scheme 1A), is a third-generation quinolone antibiotic used exclusively in veterinary medicine. It is used to treat colibacillosis and salmonellosis because of its potent antibacterial activity (1). In animals, *in vivo* studies of ENR have shown that it has good adsorption and achieves effective drug concentrations in various tissues and organs (2). In veterinary clinical practice, ENR has been approved by the FDA for use in felines and canines (3). The dosing regimens for authorized ENR tablets and injection solutions are as follows: a single oral administration of 5–20 mg/kg body weight (B. W.) and a single intramuscular administration of 2.5 mg/kg B. W., administered twice daily for 2–3 days, respectively (4). It is often necessary to maintain effective plasma concentrations with repeated dosages because of ENR’s poor aqueous solubility (0.23 g/L) (5, 6) and low bioavailability after administration (7). This problem leads to repeated stress, drug-induced side effects, the development of drug resistance, and antibiotic pollution in the environment (8, 9). Therefore, a novel formulation technology with high solubility and bioavailability is required.

Various technologies have been developed to improve drug solubility, including crystal engineering (10), solid dispersion (11), liposomes (12), nanoemulsions (13), micelles, and hydrogels (14). Among them, polymeric micelles can form a new drug delivery system through the self-assembly of amphiphilic polymers in aqueous media. The hydrophobic core of polymeric micelles can improve the solubility of hydrophobic drugs, while the hydrophilic shell protects the encapsulated drug from enzymatic degradation in biological fluids (15). In addition, the hydrated layer formed by the hydrophilic shell creates a steric barrier that hinders plasma protein adsorption onto the micelle surface, thereby reducing phagocytic clearance by immune cells (16). Polyethylene glycol (PEG) is a hydrophilic polymer segment that is generally recognized for its good biocompatibility, high water solubility, and long circulation time following intravenous administration (17). Meanwhile, poly-L-lactic acid (PLLA), a typical hydrophobic polymer segment, has various advantages, such as high biocompatibility, non-toxicity, and good biodegradability (18, 19). Preparing polymeric micelles using methoxy poly (ethylene glycol)-poly(lactide) (mPEG-PLLA) as a carrier has numerous advantages, such as biodegradability, biocompatibility, a long circulation time in the body, structural stability, and a simple preparation method (20–22). Therefore, it has been widely applied as a nanocarrier in the drug delivery system. For instance, pyrinezolid (PZ) micelles exhibit extended blood circulation time and enhanced oral bioavailability. In addition, PZ micelles boost drug exposure in the lungs while reducing it in the liver and kidneys. This suggests that PZ micelles could enhance *in-vivo* efficacy for patients with MRSA-related pneumonia and reduce potential renal and hepatic toxicities (18). In this study, ENR polymeric micelles (ENR-m) were prepared using the solvent evaporation method with mPEG-PLLA as the carrier (Scheme 1B). Several characterization methods were performed to characterize the ENR-m. The ENR release behavior of ENR-m *in vitro* and *in vivo* and its antibacterial activity were also evaluated.

## 2 Materials and methods

### 2.1 Materials

Enrofloxacin (ENR, purity ≥ 98%) was procured from Aladdin Biochemical Technology Co., Ltd. (Shanghai, China), while Enrofloxacin Standard (purity ≥ 98%) was obtained from Solarbio Science & Technology Co., Ltd. (Beijing, China). ENR commercially available tablets were obtained from Hebei Kexing Pharmaceutical Co., Ltd. (Shijiazhuang, China). mPEG-PLLA was sourced from Daigang Biomaterial Co., Ltd. (Jinan, China). Acetone came from Kemiou Chemical Reagent Co., Ltd. (Tianjin, China), and high - performance liquid chromatography (HPLC) - grade acetonitrile was purchased from Thermo Fisher Scientific (Waltham, USA). All other chemicals and solvents of analytical grade were commercially acquired.

*Escherichia coli* (*E. coli*) and *Salmonella typhi* (*S. typhi*) strain were sourced from China General Microbiological Culture Collection Center (Beijing, China). Healthy male beagles (weighing 11–16 kg) were obtained from Marshall Biotechnology Co., Ltd. (Beijing, China).

### 2.2 Preparation of ENR polymeric micelles (ENR-m)

ENR-m were prepared using the solvent evaporation method, as described previously (21), with appropriate modifications. In this method, mPEG-PLLA (3 mg) and ENR (6 mg) were dissolved in 3 mL of acetone (oil phase) using an ultrasonicator (SB5200DT, Ningbo Scientz Biotechnology Co., Ltd., China). The mixture was injected into water (aqueous phase) at a rate of 0.3 mL/min using a syringe pump (LSP01-1A, Baoding Ditron electronic technology Co., Ltd., China), followed by solvent evaporation with a rotary evaporator (RE-52AA, Shanghai Yarong Biochemical Instrument Co., Ltd., China). After complete evaporation, a dried film was obtained. The film was then hydrated with 15 mL of ultrapure water. Finally, the mixture was filtered to remove excessive unencapsulated drug and stored at 4°C for subsequent analysis.

### 2.3 Drug loading (DL, %) and entrapment efficiency (EE, %)

The DL and EE of the ENR-m formulations were measured using the centrifugation technique described in another report (23). In brief, 1 mL of the ENR-m was centrifuged at 12000 rpm for 10 min to obtain the supernatant. The supernatant was diluted with the mobile phase, and it was demulsified by ultrasound for 10 min. The amount of ENR in the micelles was determined by HPLC at 273 nm. The DL (%) and EE (%) values were determined using the equations described in reference (24), and each sample was assayed in triplicate. The DL (%) and EE (%) of the ENR-m were calculated using the following Equations 1, 2.


(1) $$\mathrm{DL}\;(\%)=\frac{\text{amount of drug in the micelles}}{\text{total micelles weight}\;(\text{drug}+\text{polymer})}\times 100\%$$

(2) $$\mathrm{EE}\;(\%)=\frac{\text{amount of drug in the micelles}}{\text{total amount of drug initially added}}\times 100\%$$

### 2.4 Screening and optimization of ENR micelles using a Box–Behnken design (BBD)

A three-factor, three-level BBD was employed to optimize the ENR-m. As shown in Supplementary Table S1, three main components were included as independent variables, along with their low, medium, and high levels. DL (%) and EE (%) were used as dependent responses to determine the optimal formulation conditions. Subsequently, Design-Expert® (version 13.0.1.0, State Ease Inc., Minneapolis, MN, USA) was used to generate a 17-run BBD. The optimized blank and ENR micelles were lyophilized at −80°C for pending further analysis.

### 2.5 Characterization of the ENR-m

#### 2.5.1 Determination of particle size (PS) and the polydispersity index (PDI)

The PS and PDI of the blank micelles and ENR-m were measured in triplicate by dynamic light scattering (DLS) using a Malvern Zetasizer Nano (ZS90, Malvern Instruments Ltd., UK). Each sample was equilibrated at 25°C for 2 min before analysis.

#### 2.5.2 Transmission electron microscopy (TEM)

The morphology of the blank micelles and ENR-m was observed using TEM (Talos L120C, Thermo Fisher Scientific Inc. USA). One drop of the blank micelle and ENR-m solutions was, respectively, placed on a copper grid. After 1 min, the surface water was removed, and a drop of 2 wt% phosphotungstic acid was immediately added. The samples were observed after air drying.

#### 2.5.3 Fourier transform infrared spectroscopy (FT-IR)

The FT-IR spectrum was collected using an ALPHA FT-IR spectrometer (Bruker, Germany) with KBr pellets. Scanning was performed over a wavenumber range of 4,000–400 cm^−1^ at a resolution of 0.1 cm^−1^.

### 2.6 Accelerated stability test

To assess storage stability, accelerated stability environment testing of the ENR-m was performed. Briefly, ENR-m powders were stored at 40°C. A relative humidity (RH) of 75% was achieved within the stability chamber. An appropriate amount of the ENR-m was taken at 0, 15, 30, and 60 days, and the ENR content was determined by HPLC. DL (%) and EE (%) were used as key indicators.

### 2.7 *In vitro* drug release study

The *in vitro* drug release behavior of ENR and the ENR-m was studied using the dialysis method (25) as follows: the ENR-m (5 mL) and ENR (4.5 mL, equivalent to 5 mL of the ENR-m) were placed in a dialysis bag with a molecular weight cutoff of 12,000 Daltons. The dialysis bag was incubated in 300 mL of phosphate buffer (37°C, pH 6.8) with shaking at approximately 100 rpm. At the predetermined time intervals, 1 mL of the solution was withdrawn from the release medium, and an equal volume of fresh medium was added to maintain the sink condition. The ENR content was quantitatively determined using UV–Vis spectrophotometry (UV-2450, Shimadzu, Japan) at 273 nm. The cumulative release rate (Q%) of ENR and the ENR-m was calculated using the following equation:


$$(Q\%)=\frac{V_{0}C_{n}+V_{i}\sum_{1}^{n-1}C_{i}}{W}\times 100\%$$

In the above equation, V_0_ is the total volume of the release medium (mL); C_n_ is the concentration of enrofloxacin (mg/mL) measured during n time sampling; V_i_ is the volume of each sampling (mL); C_i_ is the concentration of enrofloxacin at i time point (mg/mL); W is the total content of enrofloxacin (mg).

### 2.8 Pharmacokinetics study

A pharmacokinetic study was conducted based on previous research from our laboratory (26). A total of nine healthy male beagles were housed in an environment-controlled room with access to fresh food and water, and they were then randomly divided into three groups (*n* = 3). The powder samples were suspended in 0.5% CMC-Na, and then the uniform drug suspension was orally administrated to the beagles as a single dose of 10 mg/kg pure ENR, 11.11 mg/kg ENR-m, and 10.42 mg/kg commercial ENR tablets. At 0.083, 0.167, 0.25, 0.583, 0.75, 1, 1.5, 2, 4, 6, 8, 2, and 24 h post-administration, 1 mL of blood was drawn from the forelimb vein. All blood samples were transferred to heparin sodium-anticoagulated tubes and centrifuged at 6000 rpm for 10 min. The resulting supernatant was then collected and stored at −20°C for subsequent analysis. The animal experimental protocol and procedures used in this study were approved by the Animal Care and Use Committee of Hebei Agricultural University (Baoding, China; protocol number 2021058; approval date 28 February 2021) and were conducted in accordance with relevant guidelines and regulations.

Furthermore, 100 μL of plasma was combined with 300 μL of methanol. The mixture was vortexed for 3 min and centrifuged at 12000 rpm for 10 min. Subsequently, the supernatant was filtered and analyzed using HPLC. DAS 2.0 software (Mathematical Pharmacology Professional Committee of China, Shanghai, China) was used to calculate key pharmacokinetic parameters using the non-compartment method. These parameters included the maximum ENR plasma concentration (C_max_), time to reach C_max_ (T_max_), half-life (t_1/2_), and the area under the ENR plasma concentration-time curve (AUC). One-way analysis of variance was performed using SPSS 19.0 to evaluate the significance of differences between the groups.

### 2.9 HPLC analysis

The ENR concentration was determined using an HPLC system (Model 1,525, Waters Corporation, MA, U. S. A). The setup included a Waters 2,998 PDA detector and a Waters C18 column (5 μm, 4.6 mm × 250 mm). The column was kept at 37°C. The mobile phase, a mixture of 0.025 M aqueous phosphoric acid (pH 2.5 ± 0.1 with triethylamine) and acetonitrile (82:18, *v/v*), was pumped at a flow rate of 1 mL/min. A total of 20 μL of the sample was injected for analysis.

### 2.10 *In vitro* antibacterial study

The agar diffusion method was employed to observe the antibacterial activity of ENR and the ENR-m against *E. coli* and *S. typhi* (27). All bacterial strains were cultivated in nutrient broth (Hopebiol Ltd., Shandong, China) and incubated at 37°C for 24 h. A bacterial suspension of each strain (100 μL) was inoculated onto nutrient agar. On each inoculated plate, four stainless steel cylinders (F6621, Beyotime Institute of Biotechnology, Shanghai, China) of the same size (8 × 6 × 10 mm) were placed. ENR and the ENR-m were, respectively, dissolved in NaOH (0.1 mol/L) and sterile water, and the cylinders were filled with the sample solutions (2.5, 5, and 10 μg/mL). NaOH (0.1 mol/L) and sterile water were used as control groups, respectively. The agar plates were incubated at 37°C for 24 h, and a digital caliper was used to measure the diameters of the growth inhibition zones.

## 3 Results

### 3.1 Formulation of ENR-m

According to the single-factor experiments, EE (%) and DL (%) showed significant variations with changes in the concentrations of mPEG-PLLA, the water-to-oil ratio, and the feed ratio. In the BBD experiment, a total of 17 runs were conducted to optimize the formulation. The concentration of mPEG-PLLA (A) ranged from 0.1 to 3 mg/mL, the water-to-oil ratio (B) ranged from 10:2 to 10:4, and the feed ratio (mPEG-PLLA: ENR; C) ranged from 1:1 to 1:3. DL (Y1) and EE (Y2) were used as dependent variables (responses). Supplementary Table S2 displays the results of the BBD experiments conducted to evaluate the three independent variables.

The results in Supplementary Tables S3, S4 present the analysis of variance for the two responses. The lack-of-fit test for DL (%) resulted in *p* = 0.3321 and R^2^ = 0.9971, while for EE (%), the values were *p* = 0.4207 (*p* > 0.05) and R^2^ = 0.9950. This indicated an excellent goodness of fit at *p* < 0.0001. The coefficients of variation for DL (%) and EE (%) were 0.87 and 2.48, respectively.

**Figure 1:**
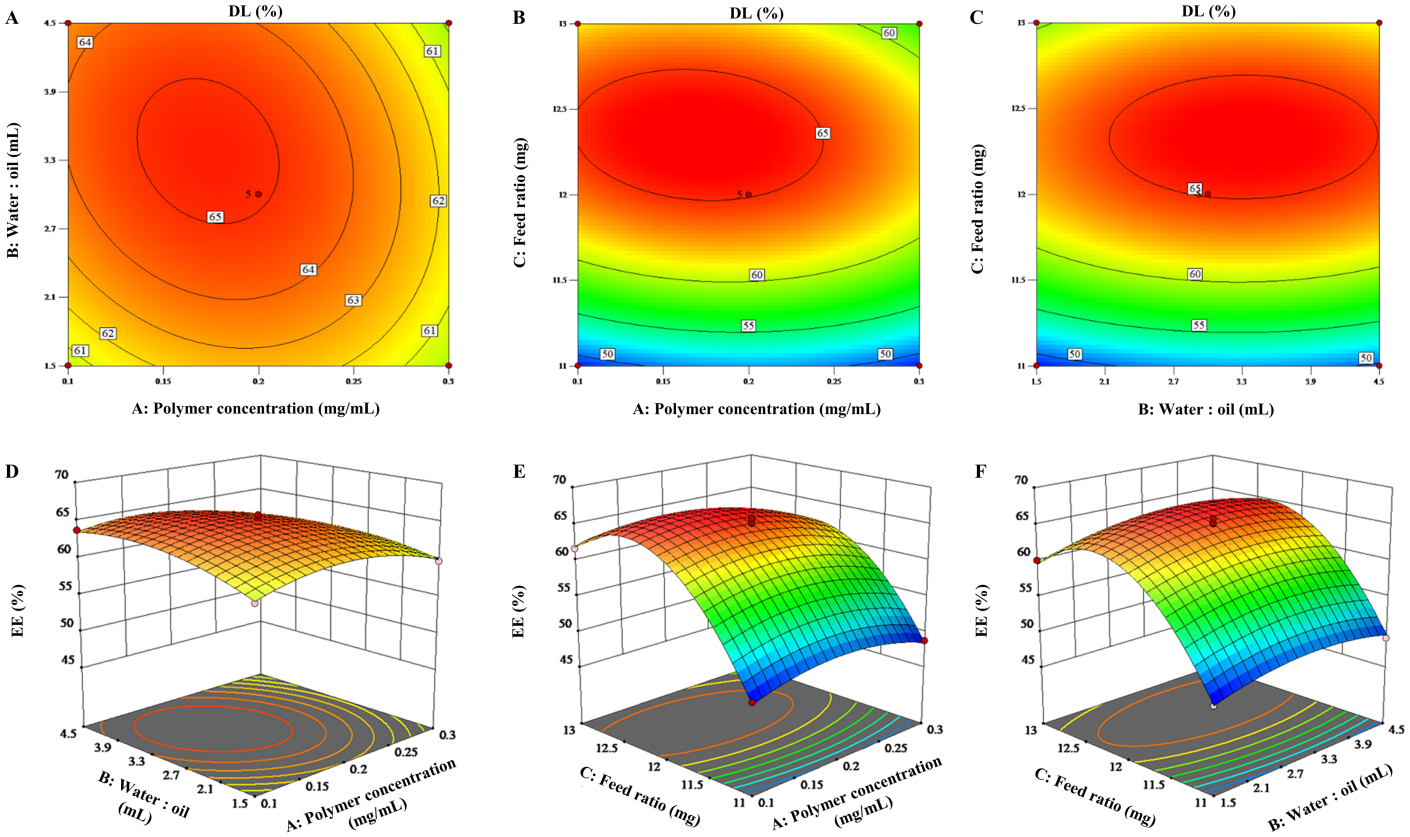
3D response surface graphs **(A–C)** and 2D contour plots **(D–F)** showing the effects of the different factors on the drug loading content of the ENR-m (%).

**Figure 2:**
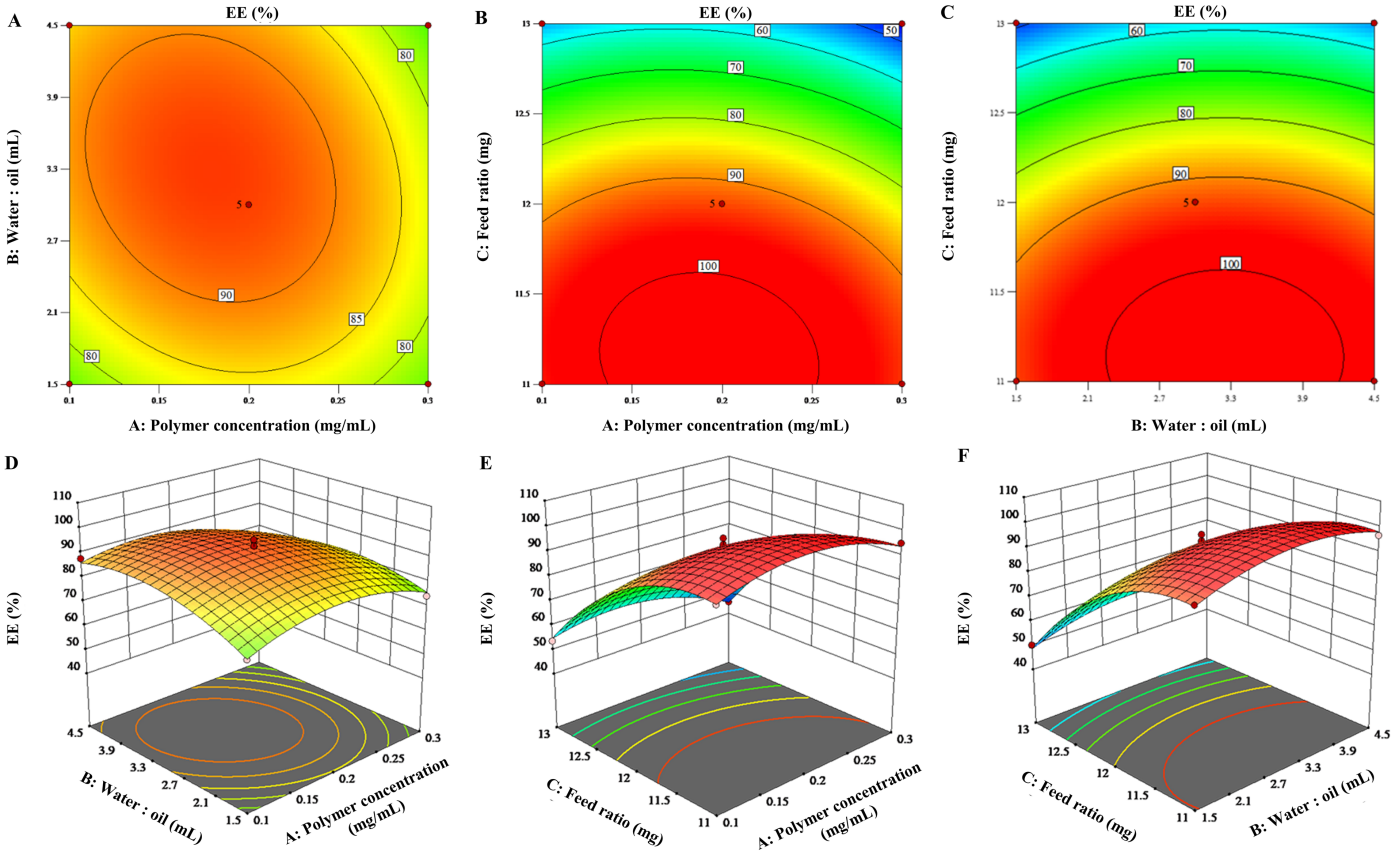
3D response surface graphs **(A–C)** and 2D contour plots **(D–F)** showing the effects of the different factors on the encapsulation efficiency of the ENR-m (%).

To visualize the effects of the three factors on the drug loading of *the* ENR-m, response surface analysis was conducted to describe the regression equations that explain the relationship between these factors and the response. Both 2D contour plots and 3D response *surface* graphs were generated to assess the impact of single and multiple factors on the response (28). As depicted in Figures 1A–C, 2A–C, the 3D response surface graphs show the significant effects of the polymer concentration (A), water-to-oil ratio (B), and feed ratio (C) on the response of DL (%) and EE (%). This is also observed in the corresponding 2D contour plots (Figures 1D–F, 2D–F), which illustrate how the three factors worked together and interacted with each other to influence changes in DL (%) and EE (%).

By analyzing the 2D contour plots and 3D response surface graphs, the optimal formulation conditions were obtained as follows: mPEG-PLLA concentration of 0.17 mg/mL, water-to-oil ratio of 15:3.4 (w/w), and feed ratio of 1:2.4 (w/w). The optimized formulation was prepared. Then, the experimental results were compared with the predicted ones to validate the optimization process. As a result, the predicted values of DL (%) and EE (%) were 68.38 ± 0.22% and 88.40 ± 0.91%, respectively, in the calculated model, which showed no significant difference from the experimental values (*p* > 0.05). Thus, the BBD optimization of the ENR-m was adequately validated.

### 3.2 Dynamic light scattering (DLS) and transmission electron microscopy (TEM)

**Figure 3:**
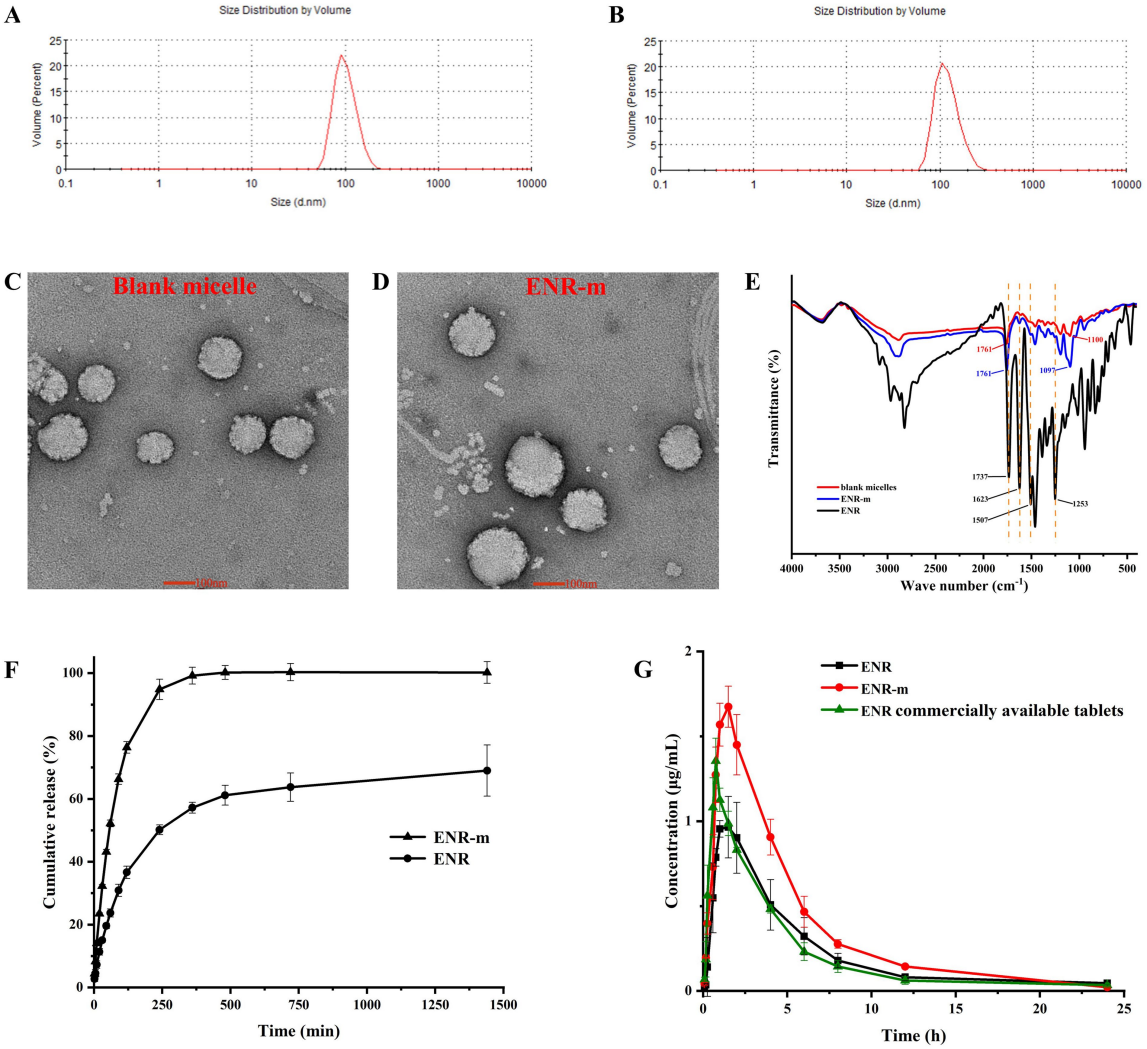
Characterization of the ENR-m and improved physicochemical properties of ENR. **(A,B)** The particle size distribution of the blank micelles and ENR-m; **(C,D)** TEM images of the blank micelle and ENR-m; **(E)** FT-IR spectra of ENR, the blank micelles, and the ENR-m; **(F)** *In vitro* release profiles of ENR and the ENR-m. **(G)** Plasma concentration-time profiles of ENR, the ENR-m, and the commercially available tablets after oral administration at 10 mg/kg in the beagles. (mean ± SD, *n* = 3).

The particle sizes of blank micelles and ENR-m were determined using DLS. The results showed (Supplementary Table S5 and Figures 3A,B) that the average particle sizes of blank micelles and ENR-m were 109.03 ± 4.29 nm and 133.67 ± 3.10 nm, respectively, with an excellent PDI of 0.11 ± 0.07 and 0.13 ± 0.03. The results indicated that ENR-m had a homogeneous micelle system because of the low PDI value (<0.3) (29). The average size of micelles was slightly greater after ENR loading (from 109.03 nm to 133.67 nm).

The blank micelle and ENR-m were found to possess a spherical appearance with smooth surfaces, as shown by the TEM images (Figures 3C,D), which correlated well with the narrow particle size distribution. Spherical particles were found with no obvious aggregation. The particle sizes of the blank micelle and ENR-m were approximately 100 nm and 120 nm, respectively.

### 3.3 Fourier transform infrared spectroscopy (FT-IR)

The FT-IR spectra of ENR, blank micelle, and ENR-m are provided in Figure 3E. The characteristic peaks of ENR appeared at 1736 cm^−1^ (C=O stretching), 1,623 cm^−1^ (C=O stretching of the pyridine group), 1,507 cm^−1^ (benzene group), and 1,252 cm^−1^ (C-F stretching of the benzene group) (30). The absorption peak from 3,000 to 2,700 cm^−1^ was assigned to the stretching of methyl and methylene groups (31). In the blank micelles, strong characteristic peaks at 1760^−1^ and 1,100 cm^−1^ corresponded to the C=O stretching of the carboxyl group (a characteristic peak of the PLLA polymer segment) and the C–O stretching of the ester linkage in the polymer, respectively (32, 33). The broad absorption band observed between 4,000 and 3,500 cm^−1^ in ENR and the blank micelles was assigned to the stretching of the O-H group. After forming ENR-m, strong characteristic peaks were observed at 1760 cm^−1^ (C=O stretching from mPEG-PLLA), 1,623 cm^−1^ (C=O stretching of pyridine rings from ENR), 1,507 cm^−1^ (C-F stretching of the benzene ring from ENR), 1,252 cm^−1^ (C-F stretching of the benzene ring from ENR), and 1,097 cm^−1^ (C-O stretching of the ester linkage from mPEG-PLLA).

### 3.4 Stability of ENR micelles

The accelerated stability of ENR micelles was evaluated at 40°C and 75% RH, and the major evaluation indicators were DL (%) and EE (%). As shown in Table 1, the DL (%) and EE (%) of ENR-m showed no significant changes over 0–60 days (*p* > 0.05). It revealed that ENR-m exhibited excellent chemical stability under environmental conditions of high temperature and humidity and could be stored for at least 60 days.

**Table 1:** DL (%) and EE (%) of the ENR-m immediately after the preparation of the micelles (0 days) and after 15, 30, and 60 days.

Time (day)	DL (%)	EE (%)
0	68.19 ± 0.07^ns^	88.02 ± 0.31^ns^
15	67.24 ± 1.19^ns^	86.57 ± 1.53^ns^
30	66.74 ± 1.08^ns^	85.93 ± 1.39^ns^
60	66.81 ± 0.43^ns^	86.02 ± 0.56^ns^

ns, no significant difference (*p* > 0.05).

### 3.5 *In vitro* drug release study

The *in vitro* release profile of ENR and ENR-m in the PBS solution (pH 6.8) is shown in Figure 3F. The cumulative release of ENR was approximately 57% at 6 h and 69% at 24 h. Pure ENR exhibited extremely low cumulative release and released only 69% over 24 h of the study, while the ENR-m released 100% within 6 h in pH 6.8 PBS. The results indicate that the solubility and *in vitro* release performance of ENR were improved by the ENR-m.

### 3.6 Pharmacokinetics study

The mean plasma concentration-time curves of pure ENR, ENR-m, and commercial ENR tablets are shown in Figure 3G, and the pharmacokinetic parameters calculated using the non-compartment model are shown in Table 2. The commercial ENR tablet and pure ENR showed similar peak plasma concentrations and bioavailability. The C_max_ of ENR in the ENR-m was approximately 165% higher than that of pure ENR. Based on the values of AUC_0–24_, the bioavailability of ENR from the ENR-m was approximately 160% greater than that of pure ENR.

**Table 2:** Pharmacokinetic parameters of ENR, ENR-m, and commercially available tablets in beagles after oral administration (mean ± SD, *n* = 3).

Drugs	T_1/2_ (h)	T_max_ (h)	C_max_ (μg/mL)	AUC_0-24_ (μg/mL·h)
ENR	3.03 ± 0.22	1.17 ± 0.29	1.04 ± 0.11	5.41 ± 1.20
ENR-m	4.38 ± 0.17^**^	1.33 ± 0.29^*^	1.72 ± 0.05^*^	8.64 ± 0.52^**^
Commercially available tablets	3.14 ± 0.56^##^	0.75 ± 0.00^*##^	1.35 ± 0.13^*##^	5.19 ± 0.34^##^

ns, *p* > 0.05. Compared with ENR: *p < 0.05 and **p < 0.01. Compared with ENR-m: ^#^*p* < 0.05 and ^##^*p* < 0.01.

### 3.7 *In vitro* antibacterial study

**Figure 4:**
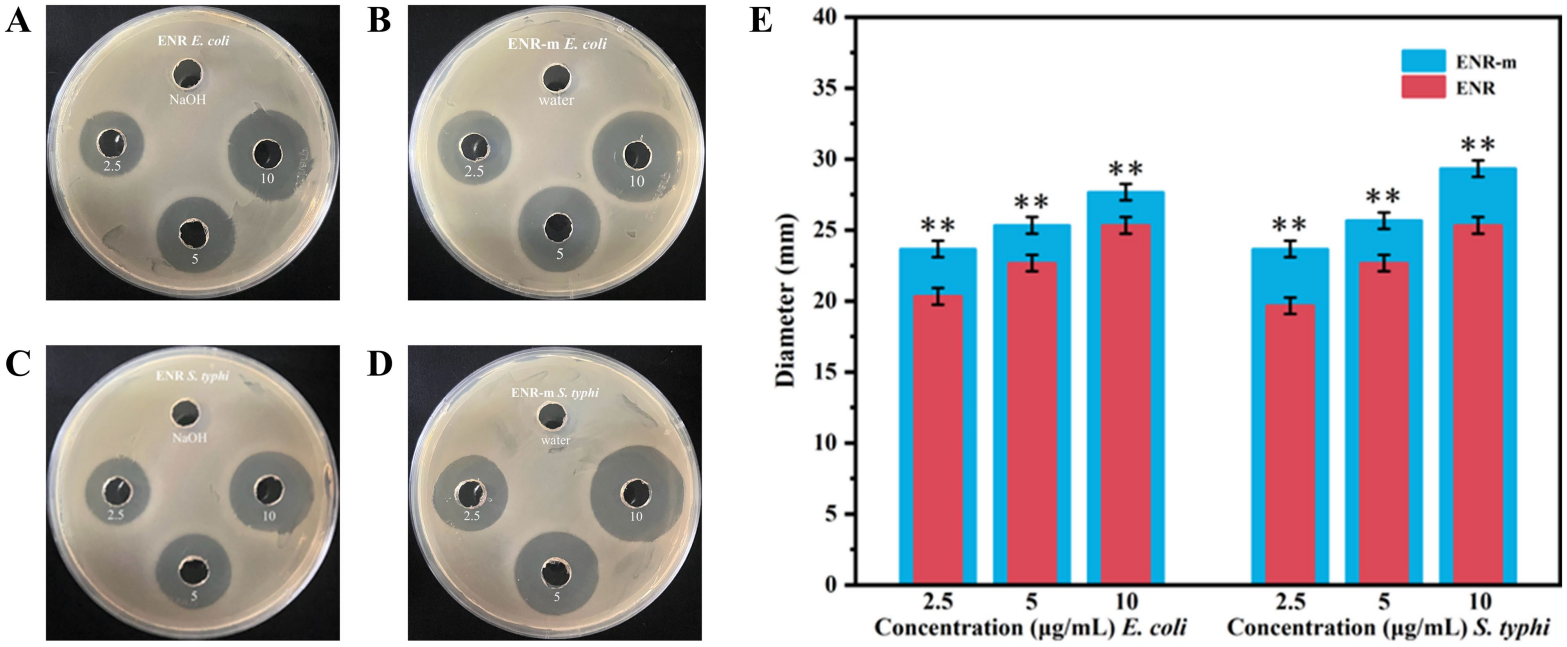
*In vitro* antibacterial activity of the ENR-m. **(A,B)** Inhibition zones of ENR and ENR-m against *E. coli*; **(C,D)** Inhibition zones of ENR and the ENR-m against *S. typhi*; **(E)** Diameter of the inhibition zones of ENR and the ENR-m on *E. coli* and *S. typhi* (compared with the ENR group: *p < 0.05 and **p < 0.01).

The antibacterial activities of ENR and ENR-m against *E. coli* and *S. typhi* were determined using the agar diffusion method. The diameter of the inhibition zone was used to evaluate the strength of the antibacterial activity. The results are shown in Figures 4A–D, and the values of the inhibition zone diameters are presented in Supplementary Table S6. Around the agar wells inoculated with *E. coli* and *S. typhi*, the clear inhibition zones produced by the ENR-m were larger than those of the pure ENR and the control groups at the same concentration. As shown in Figure 4E, compared to ENR, the *in vitro* antibacterial activity of ENR-m against *E. coli* and *S. typhi* was significantly enhanced (*p* < 0.01). For *E. coli*, the inhibition zones of ENR-m increased by 16.43, 10.13, and 7.79% at concentrations of 2.5, 5, and 10 μg/mL, respectively, compared to those of pure ENR. Similarly, for *S. typhi*, the inhibition zones of ENR-m increased by 18.35, 13.23, and 14.49% at concentrations of 2.5, 5, and 10 μg/mL, respectively, compared to those of pure ENR.

## 4 Discussion

The Box–Behnken design is one of the methods used for the experimental design of pharmaceutical formulations based on response surface methodology, characterized by extremely strong symmetry and rotatability (34). The combination of single-factor experiments and the Box–Behnken design is widely used in pharmaceutical science research (35). Goo et al. (36) developed revaprazan supersaturable micelles using a Box–Behnken design with three independent variables. This approach has great potential for the development of solidified formulations of poorly water-soluble drugs with improved oral absorption. In our Box–Behnken results, all parameters were entirely consistent with the required range for model establishment (37). Thus, the model has high predictive ability for encapsulation efficiency and drug loading of ENR-m when various factors change. Notably, when the concentrations of ENR remained unchanged, both DL (%) and EE (%) increased continuously with increasing concentrations of mPEG-PLLA. This is because mPEG-PLLA maintains the stability of the system by forming micelles when its concentration surpasses the critical micelle concentration. In addition, increased hydrophobic space and hydrophobic interactions can serve as driving forces for the formation of polymeric micelles (38). Accordingly, a larger number of ENR molecules can be efficiently incorporated into the micellar core. When the concentration of mPEG-PLLA remained unchanged, DL (%) initially decreased and then increased asthe ENR content increased. The drug in the hydrophobic core does not reach saturation when the ENR content is low. However, the chance of collisions and agglomeration between drug molecules and polymer increases when the drug dosage increases, allowing the excess drug to recombine into large drug particles (39). The rate of ENR combination is significantly faster than the encapsulation process by the micelle core, leading to a decrease in drug loading (40). In summary, hydrophobic interactions between the micelles core and the drug and the precipitation of hydrophobic drugs are competitive processes. In other words, drug molecules at lower dosages can be effectively encapsulated into the micelle core, but drug loading remains low. However, excessive dosages lead to aggregation of drug particles, which can also lead to a decrease in drug loading.

As mentioned, the drug release of fluoroquinolone antimicrobials is strongly influenced by the pH of the environment, and the studied drugs exist as poorly soluble zwitterionic molecules in the natural environment (41). According to the Noyes*–*Whitney equation, the dissolution rate of a solute is determined by the surface area of the solute particles, the diffusion coefficient, the thickness of the concentration gradient, the solute concentration at the particle surface (saturation concentration), and the solute concentration in the bulk solvent/solution (42). If the particle surface of the solute exhibits different saturated concentrations in different solvents, then the dissolution rates of the solute will also vary in these different solvents. For example, the release performance of *v*itexin (Vi) was studied in an HCl solution (pH 1.2) and PBS solution (pH 6.8 and pH 7.4). The equilibrium solubility of pure Vi was much higher at pH 7.4 compared to pH 1.2 and pH 6.8 (equilibrium solubility was 32.53 μg/mL, 60.32 μg/mL, and 121.49 μg/mL, respectively). Therefore, the cumulative release at pH 7.4 (40%) was significantly better compared to pH 1.2 (26%) and pH 6.8 (26%) (25). In addition, the concentration difference between the nanoparticles and dissolved medium can also improve the release of drugs. In summary, ENR-m may promote the release of ENR by increasing its solubility.

Among all pharmacokinetic parameters, the ENR-m showed higher peak plasma concentrations and bioavailability, consistent with results previously reported in rats (43). First, polymeric micelles can improve the solubility of hydrophobic drugs, thereby enhancing their bioavailability (44). Secondly, mPEG-PLLA is a typical amphiphilic block copolymer, possessing excellent micelle-forming ability and drug-releasing performance. Micelles are more easily absorbed by cells into the cell membrane due to the change in surface charge from negative to neutral, facilitated by the PEG shell (18, 45–47). Therefore, mPEG-PLLA plays an important role in improving the bioavailability of ENR-m. Finally, a narrow particle size distribution and smaller particle size can extend the retention time of micelles and ENR in plasma by reducing non-selective clearance of the reticuloendothelial system (48, 49). ENR-m can penetrate the mucus layer due to its particle size advantage and increase biological adhesion to the intestinal wall through its larger specific surface area, which helps prevent rapid elimination and thereby promotes absorption (25).

In our study, we found that ENR-m had a very significant inhibitory effect on *E. coli* and *S. typhi*. However, previous research has shown that mPEG-PLLA has no antibacterial effect (50). Thus, the enhanced antibacterial effect of ENR-m may be achieved by increasing the solubility of ENR. In addition, ENR-m enter cells via endocytosis and exists within endosomes, and these endosomes can fuse with lysosomes, causing micelle depolymerization and subsequent drug release (51). Thus, the concentration of ENR was potentiated when the ENR-m was *ingested* by bacteria, thereby enhancing the antibacterial effect.

## 5 Conclusion

In summary, the solvent evaporation technique was successfully applied to the preparation of ENR-m with a spherical shape and uniform particle size. The optimal conditions determined by the Box–Behnken design were satisfactory in terms of EE% and DL%. The *in vitro* release results suggested a high solubility potential of the ENR-m. The pharmacokinetic results in the beagles demonstrated the improved bioavailability of the ENR-m. Compared to the pure drug, ENR-m exhibited enhanced antibacterial activity against *E. coli* and *S. typhi*. Therefore, the results of this study suggest that polymeric micelles are an efficient drug delivery system with promising potential for pharmaceutical applications.

## Data Availability

The original contributions presented in the study are included in the article/Supplementary material, further inquiries can be directed to the corresponding authors.
