# Supplementary material for: Development and evaluation of mPEG-PLLA polymeric micelles encapsulating enrofloxacin for enhanced solubility, bioavailability, and antibacterial performance
Source: Front Vet Sci. 2025 Jul 16;12:1595137. doi: 10.3389/fvets.2025.1595137 (PMC12307181; doi:10.3389/fvets.2025.1595137)
Supplement: Supplementary file 1 [file Data_Sheet_1.docx]

Development and evaluation of mPEG-PLLA polymer micelles encapsulating enrofloxacin for enhanced solubility, bioavailability, and antibacterial performance

Yanling Sun^a^, Yanan Maoa, Xin Hea^*^, Xinghua Zhaoa^*^

^a^ College of Veterinary Medicine, Hebei Agricultural University, Baoding, 071000.

**Table S1** Independent and dependent variables in Box-Behnken design for preparation and optimization of ENR-m

| Factors | Levels | | |
| --- | --- | --- | --- |
| Independent Variable | Low (-1) | Centre (0) | High (+1) |
| A=concentrations of mPEG-PLLA (mg/mL) | 0.1 | 0.2 | 0.3 |
| B= water-oil ratio | 10∶2 | 10∶3 | 10∶4 |
| C= feed ratio | 1∶1 | 1∶2 | 1∶3 |
| Dependent variable/response | Constraints | | |
| Y_1_= drug loading | Maximize | | |
| Y_2_= Entrapment efficiency | Maximize | | |

**Table S2** Experimental runs and results of responses for BBD

| Std | Run | A | B | C | DL (%) | EE (%) |
| --- | --- | --- | --- | --- | --- | --- |
| 17 | 1 | 0 | 0 | 0 | 64.72 | 91.72 |
| 3 | 2 | -1 | 1 | 0 | 63.70 | 87.75 |
| 15 | 3 | 0 | 0 | 0 | 65.79 | 96.16 |
| 2 | 4 | 1 | -1 | 0 | 59.74 | 74.21 |
| 8 | 5 | 1 | 0 | 1 | 57.59 | 45.27 |
| 16 | 6 | 0 | 0 | 0 | 64.62 | 91.34 |
| 10 | 7 | 0 | 1 | -1 | 49.11 | 96.50 |
| 7 | 8 | -1 | 0 | 1 | 61.67 | 53.64 |
| 13 | 9 | 0 | 0 | 0 | 65.09 | 93.24 |
| 4 | 10 | 1 | 1 | 0 | 60.03 | 75.11 |
| 12 | 11 | 0 | 1 | 1 | 61.28 | 52.76 |
| 9 | 12 | 0 | -1 | -1 | 48.40 | 93.80 |
| 14 | 13 | 0 | 0 | 0 | 65.19 | 93.65 |
| 6 | 14 | 1 | 0 | -1 | 48.70 | 94.95 |
| 1 | 15 | -1 | -1 | 0 | 60.22 | 75.71 |
| 5 | 16 | -1 | 0 | -1 | 48.81 | 95.36 |
| 11 | 17 | 0 | -1 | 1 | 60.09 | 50.19 |

**Table S3** Analysis of variance of drug loading for ENR-m response surface results

| Source | Sum of Squares | df | Mean Suares | *F* Value | *P*-value |
| --- | --- | --- | --- | --- | --- |
| Model | 644.60 | 9 | 71.62 | 271.07 | <0.0001 |
| A | 8.69 | 1 | 8.69 | 32.89 | 0.0007 |
| B | 4.01 | 1 | 4.01 | 15.17 | 0.0059 |
| C | 260.09 | 1 | 260.09 | 984.35 | <0.0001 |
| AB | 2.54 | 1 | 2.54 | 9.60 | 0.0174 |
| AC | 3.95 | 1 | 3.95 | 14.97 | 0.0061 |
| BC | 0.0581 | 1 | 0.0581 | 0.2199 | 0.6534 |
| A^2^ | 23.07 | 1 | 23.07 | 87.30 | <0.0001 |
| B^2^ | 13.88 | 1 | 13.88 | 52.54 | 0.0002 |
| C^2^ | 307.57 | 1 | 307.57 | 1164.06 | <0.0001 |
| Residual | 1.85 | 7 | 0.2642 |  |  |
| Lack of Fit | 0.9948 | 3 | 0.3316 | 1.55 | 0.3321 |
| Pure Error | 0.8548 | 4 | 0.2137 |  |  |
| Cor Total | 646.45 | 16 |  |  |  |

**Table S4** Analysis of Variance of encapsulation efficiency of ENR-m Response Surface Results

| Source | Sum of Squares | df | Mean Suares | *F* Value | *p*-value |
| --- | --- | --- | --- | --- | --- |
| Model | 5451.08 | 9 | 605.68 | 153.80 | <0.0001 |
| A | 65.69 | 1 | 65.69 | 16.68 | 0.0047 |
| B | 41.41 | 1 | 41.41 | 10.51 | 0.0142 |
| C | 3994.42 | 1 | 3994.42 | 1014.29 | <0.0001 |
| AB | 31.01 | 1 | 31.01 | 7.87 | 0.0263 |
| AC | 15.87 | 1 | 15.87 | 4.03 | 0.0847 |
| BC | 0.0042 | 1 | 0.0042 | 0.0011 | 0.9750 |
| A^2^ | 270.62 | 1 | 270.62 | 68.72 | <0.0001 |
| B^2^ | 206.87 | 1 | 206.87 | 52.53 | 0.0002 |
| C^2^ | 700.61 | 1 | 700.61 | 177.91 | <0.0001 |
| Residual | 27.57 | 7 | 3.94 |  |  |
| Lack of Fit | 12.89 | 3 | 4.33 | 1.19 | 0.4207 |
| Pure Error | 14.59 | 4 | 3.65 |  |  |
| Cor Total | 5478.65 | 16 |  |  |  |

**Table S5** Result of particle size and PDI for blank micelles and ENR-m

| Sample | particle size (nm) | PDI |
| --- | --- | --- |
| Blank micelles | 109.03 ± 4.29 | 0.11 ± 0.07 |
| ENR-m | 133.67 ± 3.10 | 0.13 ± 0.03 |

**Table S6** The diameter values (mm) of inhibitory zones of ENR and ENR-m on bacteria.

|  | *E. coli* (mm) | | | *S. typhi* (mm) | | |
| --- | --- | --- | --- | --- | --- | --- |
|  | 2.5 μg/mL | 5 μg/mL | 10 μg/mL | 2.5 μg/mL | 5 μg/mL | 10 μg/mL |
| ENR | 20.33±0.58 | 23.00±0.00 | 25.67±0.58 | 20.00±0.00 | 22.67±0.58 | 25.33±0.58 |
| ENR-m | 23.67±0.58 | 25.33±0.58 | 27.67±0.58 | 23.67±0.58 | 25.67±0.58 | 29.00±0.00 |

**The single-factor investigation of ENR-m formulation**

(1) The influence of types of organic solvents

The effects of different organic solvents on the drug loading and encapsulation efficiency of ENR-m prepared by the solvent evaporation method are shown in the following table. The ENR-m prepared with acetone as the organic solvent obtained the highest drug loading and encapsulation efficiency (*P* < 0.05), which were 65.10% and 93.32%, respectively.

Effect of organic solvent on drug loading and encapsulation efficiency of ENR-m (*n* = 6)

| Organic solvents | Drug loading (DL%) | Encapsulation efficiency (EE%) |
| --- | --- | --- |
| Acetonitrile | 61.94 ± 0.78^c^ | 81.41 ± 2.74^b^ |
| Methanol | 62.63 ± 0.47^b^ | 83.81 ± 1.67^b^ |
| Acetone | 65.10 ± 0.69^a^ | 93.32 ± 2.86^a^ |

Note: There is no significant difference between the same letters on the shoulder of the peer data (*P* > 0.05), the difference between different letters is significant (*P* < 0.05).

(2) The influence of concentration of mPEG-PLLA

The effects of different mPEG-PLLA concentrations on the drug loading and encapsulation efficiency of ENR-m prepared by the solvent evaporation method are shown in table as follows. With the increase of the concentration of mPEG-PLLA, both the drug loading capacity and the encapsulation efficiency of ENR-m show a trend of increasing first and then decreasing. When the polymer concentration is 0.2 mg/mL, the drug loading capacity and the encapsulation efficiency of ENR-m are the highest. They were 65.00% and 92.92%, respectively.

Effect of mPEG-PLLA concentration on drug loading and encapsulation efficiency of ENR-m (*n* = 6)

| The concentration of mPEG-PLLA | Drug loading (DL%) | Encapsulation efficiency (EE%) |
| --- | --- | --- |
| 0.05 mg/mL | 63.19 ± 0.57^c^ | 85.87 ± 2.10^c^ |
| 0.1 mg/mL | 63.88 ± 0.10^b^ | 88.42 ± 0.40^b^ |
| 0.2 mg/mL | 65.00 ± 0.74^a^ | 92.92 ± 3.02^a^ |
| 0.3 mg/mL | 60.80 ± 0.33^d^ | 77.58 ± 1.09^d^ |

Note: There is no significant difference between the same letters on the shoulder of the peer data (*P* > 0.05), the difference between different letters is significant (*P* < 0.05)

(3) The influence of water-oil ratio on encapsulation efficiency and drug loading

The effects of different water-oil ratios on the drug loading and encapsulation efficiency of ENR-m prepared by the solvent evaporation method are shown in the following table. With the increase of the water-oil ratio, both the drug loading capacity and the encapsulation efficiency of the micelles show an increasing trend. Considering that the organic solvent acetone used is an environmentally unfriendly solvent and reducing the amount of organic solvent has a relatively small impact on the drug loading and encapsulation efficiency, the optimal water-oil ratio of 10:3 is selected, and the corresponding drug loading and encapsulation efficiency are 63.53% and 87.27% respectively.

Table 5 Effect of water oil ratio on drug loading and encapsulation efficiency of ENR-m (*n* = 6)

| Water-oil ratio | Drug loading (DL%) | Encapsulation efficiency (EE%) |
| --- | --- | --- |
| 10:2 | 62.67 ± 0.72 | 83.95 ± 2.54 |
| 10:3 | 63.53 ± 1.33 | 87.27 ± 4.94 |
| 10:4 | 63.63 ± 1.33 | 87.78 ± 6.98 |
| 10:5 | 64.10 ± 0.64 | 89.32 ± 2.49 |

(4) The influence of the feed ratio

The effects of different feed ratios on the drug loading and encapsulation efficiency of ENR-m prepared by the solvent evaporation method are shown in the following table. With the increase of the feed ratio, the drug loading of ENR-m first increases and then decreases, and the encapsulation efficiency gradually decreases. When the feed ratio is 1:2, the drug loading and encapsulation efficiency of ENR-m are 63.77% and 88.01% respectively, and the comprehensive evaluation is the best.

Effect of feed ratio on drug loading and encapsulation efficiency of ENR-m (*n* = 6)

| Feed ratio | Drug loading (DL%) | Encapsulation efficiency (EE%) |
| --- | --- | --- |
| 2:1 | 33.13 ± 0.17^d^ | 99.07 ± 0.74^a^ |
| 1:1 | 49.21 ± 0.17^c^ | 96.87 ± 0.66^b^ |
| 1:2 | 63.77 ± 0.15^b^ | 88.01 ± 0.58^c^ |
| 1:3 | 62.57 ± 0.21^a^ | 55.72 ± 0.50^d^ |

Note: There is no significant difference between the same letters on the shoulder of the peer data (*P* > 0.05), the difference between different letters is significant (*P* < 0.05).

| 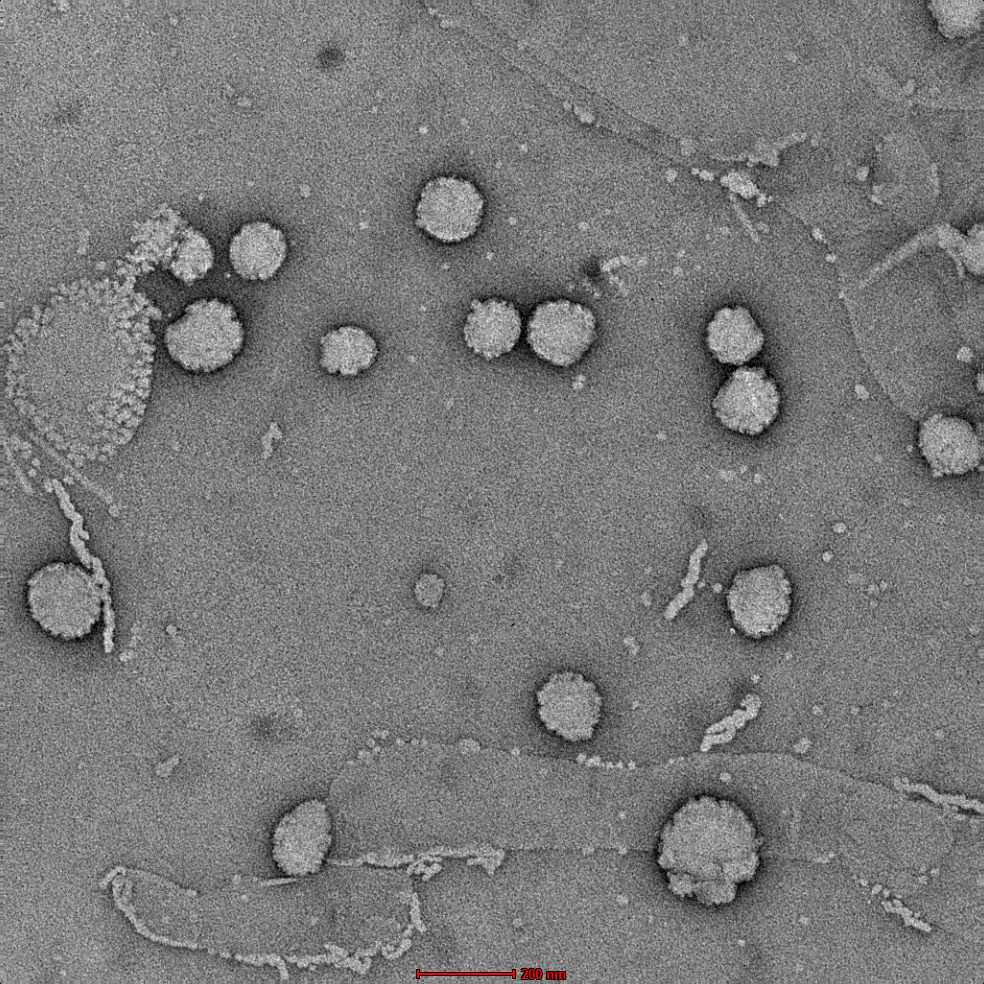  A | 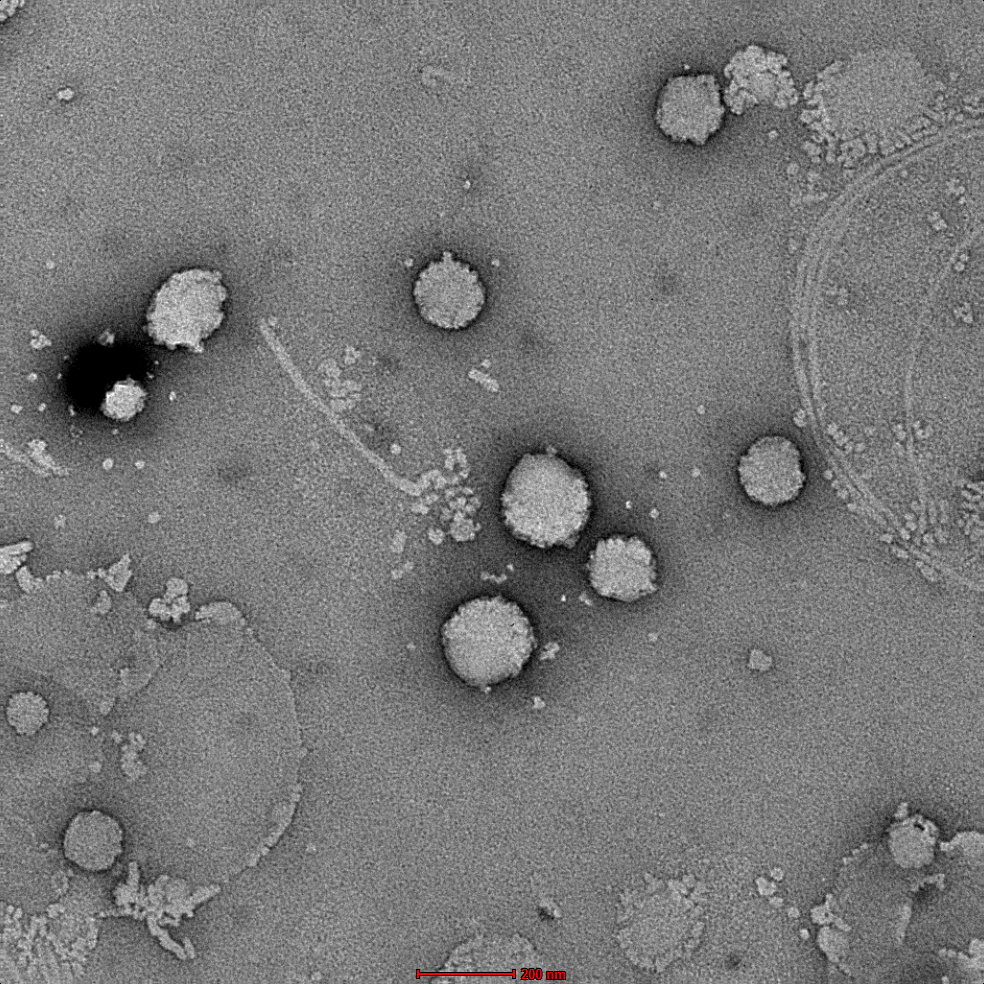  B |
| --- | --- |

Figure S1 The original TEM images of (A)ENR and (B) ENR-m.
